# Guidance to best tools and practices for systematic reviews

**DOI:** 10.11124/JBIES-23-00139

**Published:** 2023-06-07

**Authors:** Kat Kolaski, Lynne Romeiser Logan, John P.A. Ioannidis

**Affiliations:** 1 Departments of Orthopaedic Surgery, Pediatrics, and Neurology, Wake Forest School of Medicine, Winston-Salem, NC, USA; 2 Department of Physical Medicine and Rehabilitation, SUNY Upstate Medical University, Syracuse, NY, USA; 3 Departments of Medicine, of Epidemiology and Population Health, of Biomedical Data Science, and of Statistics, and Meta-Research Innovation Center at Stanford (METRICS), Stanford University School of Medicine, Stanford, CA, USA

**Keywords:** certainty of evidence, critical appraisal, methodological quality, risk of bias, systematic review

## Abstract

Data continue to accumulate indicating that many systematic reviews are methodologically flawed, biased, redundant, or uninformative. Some improvements have occurred in recent years based on empirical methods research and standardization of appraisal tools; however, many authors do not routinely or consistently apply these updated methods. In addition, guideline developers, peer reviewers, and journal editors often disregard current methodological standards. Although extensively acknowledged and explored in the methodological literature, most clinicians seem unaware of these issues and may automatically accept evidence syntheses (and clinical practice guidelines based on their conclusions) as trustworthy.

A plethora of methods and tools are recommended for the development and evaluation of evidence syntheses. It is important to understand what these are intended to do (and cannot do) and how they can be utilized. Our objective is to distill this sprawling information into a format that is understandable and readily accessible to authors, peer reviewers, and editors. In doing so, we aim to promote appreciation and understanding of the demanding science of evidence synthesis among stakeholders. We focus on well-documented deficiencies in key components of evidence syntheses to elucidate the rationale for current standards. The constructs underlying the tools developed to assess reporting, risk of bias, and methodological quality of evidence syntheses are distinguished from those involved in determining overall certainty of a body of evidence. Another important distinction is made between those tools used by authors to develop their syntheses as opposed to those used to ultimately judge their work.

Exemplar methods and research practices are described, complemented by novel pragmatic strategies to improve evidence syntheses. The latter include preferred terminology and a scheme to characterize types of research evidence. We organize best practice resources in a Concise Guide that can be widely adopted and adapted for routine implementation by authors and journals. Appropriate, informed use of these is encouraged, but we caution against their superficial application and emphasize their endorsement does not substitute for in-depth methodological training. By highlighting best practices with their rationale, we hope this guidance will inspire further evolution of methods and tools that can advance the field.

## Part 1. The state of evidence synthesis

Evidence syntheses are commonly regarded as the foundation of evidence-based medicine (EBM). They are widely accredited for providing reliable evidence and, as such, they have significantly influenced medical research and clinical practice. Despite their uptake throughout health care and ubiquity in contemporary medical literature, some important aspects of evidence syntheses are generally overlooked or not well recognized. Evidence syntheses are mostly retrospective exercises, they often depend on weak or irreparably flawed data, and they may use tools that have acknowledged or yet unrecognized limitations. They are complicated and time-consuming undertakings prone to bias and errors. Production of a good evidence synthesis requires careful preparation and high levels of organization in order to limit potential pitfalls.^1^ Many authors do not recognize the complexity of such an endeavor and the many methodological challenges they may encounter. Failure to do so is likely to result in research and resource waste.

Given their potential impact on people’s lives, it is crucial for evidence syntheses to correctly report on the current knowledge base. In order to be perceived as trustworthy, reliable demonstration of the accuracy of evidence syntheses is equally imperative.^2^ Concerns about the trustworthiness of evidence syntheses are not recent developments. From the early years when EBM first began to gain traction until more recent times when thousands of systematic reviews are published monthly,^3^ the rigor of evidence syntheses has always varied. Many systematic reviews and meta-analyses had obvious deficiencies because original methods and processes had gaps, lacked precision, and/or were not widely known. The situation has improved with empirical research concerning which methods to use and standardization of appraisal tools. However, given the geometrical increase in the number of evidence syntheses being published, a relatively larger pool of unreliable evidence syntheses is being published today.

Publication of methodological studies that critically appraise the methods used in evidence syntheses is increasing at a fast pace. This reflects the availability of tools specifically developed for this purpose.^4–6^ Yet many clinical specialties report that alarming numbers of evidence syntheses fail on these assessments. The syntheses identified report on a broad range of common conditions including, but not limited to, cancer,^7^ chronic obstructive pulmonary disease,^8^ osteoporosis,^9^ stroke,^10^ cerebral palsy,^11^ chronic low back pain,^12^ refractive error,^13^ major depression,^14^ pain,^15^ and obesity.^16,17^ The situation is even more concerning with regard to evidence syntheses included in clinical practice guidelines (CPGs).^18–20^ Astonishingly, in a sample of CPGs published in 2017–18, more than half did not apply even basic systematic methods in the evidence syntheses used to inform their recommendations.^21^

These reports, while not widely acknowledged, suggest there are pervasive problems not limited to evidence syntheses that evaluate specific kinds of interventions or include primary research of a particular study design (eg, randomized versus non-randomized).^22^ Similar concerns about the reliability of evidence syntheses have been expressed by proponents of EBM in highly circulated medical journals.^23–26^ These publications have also raised awareness about redundancy, inadequate input of statistical expertise, and deficient reporting. These issues plague primary research as well; however, there is heightened concern for the impact of these deficiencies given the critical role of evidence syntheses in policy and clinical decision-making.

### Methods and guidance to produce a reliable evidence synthesis

Several international consortiums of EBM experts and national health care organizations currently provide detailed guidance (Table 1). They draw criteria from the reporting and methodological standards of currently recommended appraisal tools, and regularly review and update their methods to reflect new information and changing needs. In addition, they endorse the Grading of Recommendations Assessment, Development and Evaluation (GRADE) system for rating the overall quality of a body of evidence.^27^ These groups typically certify or commission systematic reviews that are published in exclusive databases (eg, Cochrane, JBI) or are used to develop government or agency sponsored guidelines or health technology assessments (eg, National Institute for Health and Care Excellence [NICE], Scottish Intercollegiate Guidelines Network [SIGN], Agency for Healthcare Research and Quality [AHRQ]). They offer developers of evidence syntheses various levels of methodological advice, technical and administrative support, and editorial assistance. Use of specific protocols and checklists are required for development teams within these groups, but their online methodological resources are accessible to any potential author.

**Table 1:** Guidance for development of evidence syntheses

**International consortiums**	
Cochrane (formerly Cochrane Collaboration)	https://www.cochrane.org
JBI (formerly Joanna Briggs Institute)	https://jbi.global/
**National organizations**	
National Institute for Health and Care Excellence (NICE)—United Kingdom	https://www.nice.org.uk/
Scottish Intercollegiate Guidelines Network (SIGN) —Scotland	https://www.sign.ac.uk/
Agency for Healthcare Research and Quality (AHRQ)—United States	https://www.ahrq.gov

Notably, Cochrane is the largest single producer of evidence syntheses in biomedical research; however, these only account for 15% of the total.^28^ The World Health Organization requires Cochrane standards be used to develop evidence syntheses that inform their CPGs.^29^ Authors investigating questions of intervention effectiveness in syntheses developed for Cochrane follow the Methodological Expectations of Cochrane Intervention Reviews^30^ and undergo multi-tiered peer review.^31,32^ Several empirical evaluations have shown that Cochrane systematic reviews are of higher methodological quality compared to non-Cochrane reviews.^4,7,9,11,14,32–35^ However, some of these assessments have biases: they may be conducted by Cochrane-affiliated authors, and they sometimes use scales and tools developed and used in the Cochrane environment and by its partners. In addition, evidence syntheses published in the Cochrane database are not subject to space or word restrictions, while non-Cochrane syntheses are often limited. As a result, information that may be relevant to the critical appraisal of non-Cochrane syntheses is often removed or is relegated to online-only supplements that may not be readily or fully accessible.^28^

### Influences on the state of evidence synthesis

Many authors are familiar with the evidence syntheses produced by the leading EBM organizations, but can be intimidated by the time and effort necessary to apply their standards. Instead of following their guidance, authors may employ methods that are discouraged or outdated.^28^ Suboptimal methods described in in the literature may then be taken up by others. For example, the Newcastle-Ottawa Scale (NOS) is a commonly used tool for appraising non-randomized studies.^36^ Many authors justify their selection of this tool with reference to a publication that describes the unreliability of the NOS and recommends against its use.^37^ Obviously, the authors who cite this report for that purpose have not read it. Authors and peer reviewers have a responsibility to use reliable and accurate methods and not copycat previous citations or substandard work.^38,39^ Similar cautions may potentially extend to automation tools. These have concentrated on evidence searching^40^ and selection given how demanding it is for humans to maintain truly up-to-date evidence.^2,41^ Cochrane has deployed machine learning to identify randomized controlled trials (RCTs)^2^ and studies related to COVID-19,^42^ but such tools are not yet commonly used.^43^ The routine integration of automation tools in the development of future evidence syntheses should not displace the interpretive part of the process.

Editorials about unreliable or misleading systematic reviews highlight several of the intertwining factors that may contribute to continued publication of unreliable evidence syntheses: shortcomings and inconsistencies of the peer review process, lack of endorsement of current standards on the part of journal editors, the incentive structure of academia, industry influences, publication bias, and the lure of “predatory” journals.^44–48^ At this juncture, clarification of the extent to which each of these factors contribute remains speculative, but their impact is likely to be synergistic.

Over time, the generalized acceptance of the conclusions of systematic reviews as incontrovertible has affected trends in the dissemination and uptake of evidence. Reporting of the results of evidence syntheses and recommendations of CPGs has shifted beyond medical journals to press releases and news headlines and, more recently, to the realm of social media and influencers. The lay public and policy makers may depend on these outlets for interpreting evidence syntheses and CPGs. Unfortunately, communication to the general public often reflects intentional or non-intentional misrepresentation or “spin” of the research findings.^49–52^ News and social media outlets also tend to reduce conclusions on a body of evidence and recommendations for treatment to binary choices (eg, “do it” versus “don’t do it”) that may be assigned an actionable symbol (eg, red/green traffic lights, smiley/frowning face emoji).

### Strategies for improvement

Many authors and peer reviewers are volunteer health care professionals or trainees who lack formal training in evidence synthesis.^46,53^ Informing them about research methodology could increase the likelihood they will apply rigorous methods.^25,33,45^ We tackle this challenge, from both a theoretical and a practical perspective, by offering guidance applicable to any specialty. It is based on recent methodological research that is extensively referenced to promote self-study. However, the information presented is not intended to be substitute for committed training in evidence synthesis methodology; instead, we hope to inspire our target audience to seek such training. We also hope to inform a broader audience of clinicians and guideline developers influenced by evidence syntheses. Notably, these communities often include the same members who serve in different capacities.

In the following sections, we highlight methodological concepts and practices that may be unfamiliar, problematic, confusing, or controversial. In Part 2, we consider various types of evidence syntheses and the types of research evidence summarized by them. In Part 3, we examine some widely used (and misused) tools for the critical appraisal of systematic reviews and reporting guidelines for evidence syntheses. In Part 4, we discuss how to meet methodological conduct standards applicable to key components of systematic reviews. In Part 5, we describe the merits and caveats of rating the overall certainty of a body of evidence. Finally, in Part 6, we summarize suggested terminology, methods, and tools for development and evaluation of evidence syntheses that reflect current best practices.

## Part 2. Types of syntheses and research evidence

A good foundation for the development of evidence syntheses requires an appreciation of their various methodologies and the ability to correctly identify the types of research potentially available for inclusion in the synthesis.

### Types of evidence syntheses

Systematic reviews have historically focused on the benefits and harms of interventions; over time, various types of other systematic reviews have emerged to address the diverse information needs of clinicians, patients, and policy makers.^54^ Systematic reviews with traditional components have become defined by the different topics they assess (Table 2.1). In addition, other distinctive types of evidence syntheses have evolved, including overviews or umbrella reviews, scoping reviews, rapid reviews, and living reviews. The popularity of these has been increasing in recent years.^55–58^ A summary of the development, methods, available guidance, and indications for these unique types of evidence syntheses is available in Supplemental File 2A: http://links.lww.com/SRX/A13.

**Table 2.1:** Types of traditional systematic reviews

Review type	Topic assessed	Elements of research question (mnemonic)
Intervention^59,61^	Benefits and harms of interventions used in health care	**P**opulation, **I**ntervention, **C**omparator, **O**utcome (**PICO**)
Diagnostic test accuracy^62^	How well a diagnostic test performs in diagnosing and detecting a particular disease	**P**opulation, **I**ndex test(s), and **T**arget condition (**PIT**)
Qualitative Cochrane^63^ JBI^64^	Questions are designed to improve understanding of intervention complexity, contextual variations, implementation, and stakeholder preferences and experiences Questions inform meaningfulness and appropriateness of care and the impact of illness through documentation of stakeholder experiences, preferences, and priorities	**S**etting, **P**erspective, **I**ntervention or Phenomenon of **I**nterest, **C**omparison, **E**valuation (**SPICE**) **S**ample, **P**henomenon of **I**nterest, **D**esign, **E**valuation, **R**esearch type (**SPIDER**) **Per**spective, **S**etting, **P**henomena of interest/Problem, **E**nvironment, **C**omparison (optional), **Ti**me/timing, **F**indings (**PerSPecTIF**) **P**opulation, the Phenomena of **I**nterest, and the **Co**ntext **(PICo)**
Prognostic^65^	Probable course or future outcome(s) of people with a health problem	**P**opulation, **I**ntervention (model), **C**omparator, **O**utcomes, **T**iming, **S**etting (**PICOTS**)
Etiology and risk^66^	The relationship (association) between certain factors (eg, genetic, environmental) and the development of a disease or condition or other health outcome	**P**opulation or groups at risk, **E**xposure(s), associated **O**utcome(s) (disease, symptom, or health condition of interest), the context/location or the time period and the length of time when relevant (**PEO**)
Measurement properties^67,68^	What is the most suitable instrument to measure a construct of interest in a specific study population?	**P**opulation, **I**nstrument, **C**onstruct, **O**utcomes (**PICO**)
Prevalence and incidence^69^	The frequency, distribution and determinants of specific factors, health states or conditions in a defined population (eg, how common is a particular disease or condition in a specific group of individuals?)	Factor, disease, symptom or health **co**ndition of interest, the epidemiological indicator used to measure its frequency (prevalence, incidence), the **pop**ulation or groups at risk as well as the **co**ntext/location and time period where relevant (**CoCoPop**)

Both Cochrane^30,59^ and JBI^60^ provide methodologies for many types of evidence syntheses; they describe these with different terminology, but there is obvious overlap (Table 2.2). The majority of evidence syntheses published by Cochrane (96%) and JBI (62%) are categorized as intervention reviews. This reflects the earlier development and dissemination of their intervention review methodologies; these remain well-established^30,59,60^ as both organizations continue to focus on topics related to treatment efficacy and harms. In contrast, intervention reviews represent only about half of the total published in the general medical literature, and several non-intervention review types contribute to a significant proportion of the other half.

**Table 2.2:** Evidence syntheses published by Cochrane and JBI

Cochrane Database of Systematic Reviews^a^			*JBI Evidence Synthesis* ^b^		
Category	N	%	Category	N	%
Intervention	8572	96.3	Effectiveness	435	61.5
Diagnostic	176	1.9	Diagnostic Test Accuracy	9	1.3
Overview	64	0.7	Umbrella	4	0.6
Methodology	41	0.45	Mixed Methods	2	0.3
Qualitative	17	0.19	Qualitative	159	22.5
Prognostic	11	0.12	Prevalence and Incidence	6	0.8
Rapid	11	0.12	Etiology and Risk	7	1.0
Prototype^c^	8	0.08	Measurement Properties	3	0.4
	Total=8900		Economic	6	0.6
			Text and Opinion	1	0.14
			Scoping	43	6.0
			Comprehensive^d^	32	4.5
				Total=707	

^a^ Data from https://www.cochranelibrary.com/cdsr/reviews. Accessed 17 Sep 2022.

^b^ Data obtained via personal email communication on 18 Sep 2022 with Emilie Francis, editorial assistant, *JBI Evidence Synthesis.*

^c^ Includes the following categories: prevalence, scoping, mixed methods, and realist reviews.

^d^ This methodology is not supported in the current version of the *JBI Manual for Evidence Synthesis*.

### Types of research evidence

There is consensus on the importance of using multiple study designs in evidence syntheses; at the same time, there is a lack of agreement on methods to identify included study designs. Authors of evidence syntheses may use various taxonomies and associated algorithms to guide selection and/or classification of study designs. These tools differentiate categories of research and apply labels to individual study designs (eg, RCT, cross-sectional). A familiar example is the Design Tree endorsed by the Centre for Evidence-Based Medicine.^70^ Such tools may not be helpful to authors of evidence syntheses for multiple reasons.

Suboptimal levels of agreement and accuracy even among trained methodologists reflect challenges with the application of such tools.^71,72^ Problematic distinctions or decision points (eg, experimental or observational, controlled or uncontrolled, prospective or retrospective) and design labels (eg, cohort, case control, uncontrolled trial) have been reported.^71^ The variable application of ambiguous study design labels to non-randomized studies is common, making them especially prone to misclassification.^73^ In addition, study labels do not denote the unique design features that make different types of non-randomized studies susceptible to different biases, including those related to how the data are obtained (eg, clinical trials, disease registries, wearable devices). Given this limitation, it is important to be aware that design labels preclude the accurate assignment of non-randomized studies to a “level of evidence” in traditional hierarchies.^74^

These concerns suggest that available tools and nomenclature used to distinguish types of research evidence may not uniformly apply to biomedical research and non-health fields that utilize evidence syntheses (eg, education, economics).^75,76^ Moreover, primary research reports often do not describe study design or do so incompletely or inaccurately; thus, indexing in PubMed and other databases does not address the potential for misclassification.^77^ Yet proper identification of research evidence has implications for several key components of evidence syntheses. For example, search strategies limited by index terms using design labels or study selection based on labels applied by the authors of primary studies may cause inconsistent or unjustified study inclusions and/or exclusions.^77^ In addition, because risk of bias (RoB) tools consider attributes specific to certain types of studies and study design features, results of these assessments may be invalidated if an inappropriate tool is used. Appropriate classification of studies is also relevant for the selection of a suitable method of synthesis and interpretation of those results.

An alternative to these tools and nomenclature involves application of a few fundamental distinctions that encompass a wide range of research designs and contexts. While these distinctions are not novel, we integrate them into a practical scheme (see Figure) designed to guide authors of evidence syntheses in the basic identification of research evidence. The initial distinction is between primary and secondary studies. Primary studies are then further distinguished by: 1) the type of data reported (qualitative or quantitative); and 2) two defining design features (group or single-case and randomized or non-randomized). The different types of studies and study designs represented in the scheme are described in detail in Supplemental File 2B: http://links.lww.com/SRX/A14. It is important to conceptualize their methods as complementary as opposed to contrasting or hierarchical^78^; each offers advantages and disadvantages that determine their appropriateness for answering different kinds of research questions in an evidence synthesis.

**Figure:**
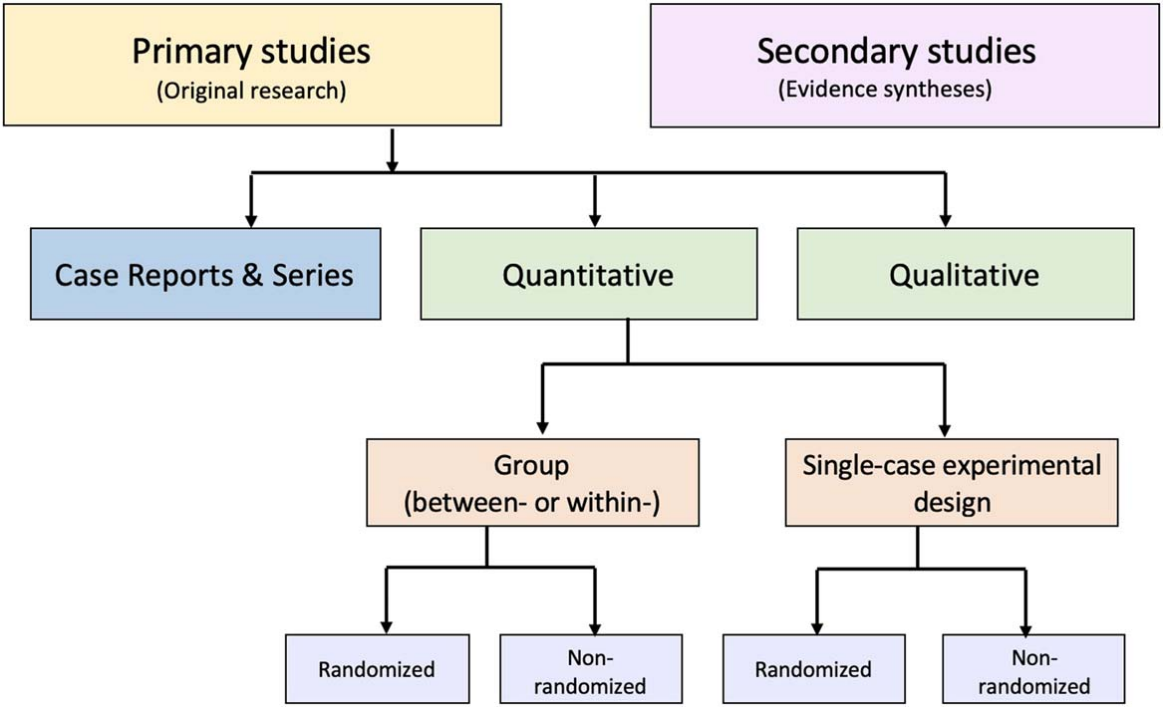
Distinguishing types of research evidence

Application of these basic distinctions may avoid some of the potential difficulties associated with study design labels and taxonomies. Nevertheless, debatable methodological issues are raised when certain types of research identified in this scheme are included in an evidence synthesis. We briefly highlight those associated with inclusion of non-randomized studies, case reports and series, and a combination of primary and secondary studies.

#### Non-randomized studies

When investigating an intervention’s effectiveness, it is important for authors to recognize the uncertainty of observed effects reported by studies with high RoB. Results of statistical analyses that include such studies need to be interpreted with caution in order to avoid misleading conclusions.^74^ Review authors may consider excluding randomized studies with high RoB from meta-analyses. Non-randomized studies of intervention (NRSI) are affected by a greater potential range of biases and thus vary more than RCTs in their ability to estimate a causal effect.^79^ If data from NRSI are synthesized in meta-analyses, it is helpful to separately report their summary estimates.^6,74^

Nonetheless, certain design features of NRSI (eg, which parts of the study were prospectively designed) may help to distinguish stronger from weaker ones. Cochrane recommends that authors of a review including NRSI focus on relevant study design features when determining eligibility criteria instead of relying on non-informative study design labels.^79,80^ This process is facilitated by a study design feature checklist; guidance on using the checklist is included with developers’ description of the tool.^73,74^ Authors collect information about these design features during data extraction and then consider it when making final study selection decisions and when performing RoB assessments of the included NRSI.

#### Case reports and case series

Correctly identified case reports and case series can contribute evidence not well captured by other designs^81^; in addition, some topics may be limited to a body of evidence that consists primarily of uncontrolled clinical observations. Murad and colleagues offer a framework for how to include case reports and series in an evidence synthesis.^82^ Distinguishing between cohort studies and case series in these syntheses is important, especially for those that rely on evidence from NRSI. Additional data obtained from studies misclassified as case series can potentially increase the confidence in effect estimates. Mathes and Pieper provide authors of evidence syntheses with specific guidance on distinguishing between cohort studies and case series, but emphasize the increased workload involved.^77^

#### Primary and secondary studies

Synthesis of combined evidence from primary and secondary studies may provide a broad perspective on the entirety of available literature on a topic. This is, in fact, the recommended strategy for scoping reviews that may include a variety of sources of evidence (eg, CPGs, popular media). However, except for scoping reviews, the synthesis of data from primary and secondary studies is discouraged unless there are strong reasons to justify doing so.

Combining primary and secondary sources of evidence is challenging for authors of other types of evidence syntheses for several reasons.^83^ Assessments of RoB for primary and secondary studies are derived from conceptually different tools, thus obfuscating the ability to make an overall RoB assessment of a combination of these study types. In addition, authors who include primary and secondary studies must devise non-standardized methods for synthesis. Note this contrasts with well-established methods available for updating existing evidence syntheses with additional data from new primary studies.^84–86^ However, a new review that synthesizes data from primary and secondary studies raises questions of validity and may unintentionally support a biased conclusion because no existing methodological guidance is currently available.^87^

### Recommendations

We suggest that journal editors require authors to identify which type of evidence synthesis they are submitting and reference the specific methodology used for its development. This will clarify the research question and methods for peer reviewers and potentially simplify the editorial process. Editors should announce this practice and include it in the instructions to authors. To decrease bias and apply correct methods, authors must also accurately identify the types of research evidence included in their syntheses.

## Part 3. Conduct and reporting

The need to develop criteria to assess the rigor of systematic reviews was recognized soon after the EBM movement began to gain international traction.^88,89^ Systematic reviews rapidly became popular, but many were very poorly conceived, conducted, and reported. These problems remain highly prevalent^23^ despite development of guidelines and tools to standardize and improve the performance and reporting of evidence syntheses.^22,28^ Table 3.1 provides some historical perspective on the evolution of tools developed specifically for the evaluation of systematic reviews, with or without meta-analysis.

**Table 3.1:** Tools specifying standards for systematic reviews with and without meta-analysis

**Reporting standards**	
Quality of Reporting of Meta-analyses (QUOROM) Statement	Moher 1999^90^
Meta-analyses Of Observational Studies in Epidemiology (MOOSE)	Stroup 2000^91^
Preferred Reporting Items for Systematic Reviews and Meta-Analyses (PRISMA) PRISMA 2020^a^	Moher 2009^92^ Page 2021^93^
**Methodological standards**	
Overview Quality Assessment Questionnaire^b^ (OQAQ)	Oxman and Guyatt 1991^94^
Systematic Review Critical Appraisal Sheet	Centre for Evidence-based Medicine 2005^95^
A Measurement Tool to Assess Systematic Reviews (AMSTAR)	Shea 2007^5^
AMSTAR-2^a^	Shea 2017^6^
**Risk of bias**	
Risk of Bias in Systematic Reviews (ROBIS)^a^	Whiting 2016^4^

^a^ Currently recommended

^b^ Validated tool for systematic reviews of interventions developed for use by authors of overviews or umbrella reviews

These tools are often interchangeably invoked when referring to the “quality” of an evidence synthesis. However, quality is a vague term that is frequently misused and misunderstood; more precisely, these tools specify different standards for evidence syntheses. Methodological standards address how well a systematic review was designed and performed.^5^ RoB assessments refer to systematic flaws or limitations in the design, conduct, or analysis of research that distort the findings of the review.^4^ Reporting standards help systematic review authors describe the methodology they used and the results of their synthesis in sufficient detail.^92^ It is essential to distinguish between these evaluations: a systematic review may be biased, it may fail to report sufficient information on essential features, or it may exhibit both problems. A thoroughly reported systematic evidence synthesis review may still be biased and flawed, while an otherwise unbiased one may suffer from deficient documentation.

We direct attention to the currently recommended tools listed in Table 3.1 but concentrate on AMSTAR-2 (update of AMSTAR [A Measurement Tool to Assess Systematic Reviews]) and ROBIS (Risk of Bias in Systematic Reviews), which evaluate methodological quality and RoB, respectively. For comparison and completeness, we include PRISMA 2020 (update of the 2009 Preferred Reporting Items for Systematic Reviews of Meta-Analyses statement), which offers guidance on reporting standards. The exclusive focus on these three tools is by design; it addresses concerns related to the considerable variability in tools used for the evaluation of systematic reviews.^28,88,96,97^ We highlight the underlying constructs these tools were designed to assess, then describe their components and applications. Their known (or potential) uptake and impact and limitations are also discussed.

### Evaluation of conduct

#### Development

AMSTAR^5^ was in use for a decade prior to the 2017 publication of AMSTAR-2; both provide a broad evaluation of methodological quality of intervention systematic reviews, including flaws arising through poor conduct of the review.^6^ ROBIS, published in 2016, was developed to specifically assess RoB introduced by the conduct of the review; it is applicable to systematic reviews of interventions and several other types of reviews.^4^ Both tools reflect a shift to a domain-based approach as opposed to generic quality checklists. There are a few items unique to each tool; however, similarities between items have been demonstrated.^98,99^ AMSTAR-2 and ROBIS are recommended for use by: 1) authors of overviews or umbrella reviews and CPGs to evaluate systematic reviews considered as evidence; 2) authors of methodological research studies to appraise included systematic reviews; and 3) peer reviewers for appraisal of submitted systematic review manuscripts. For authors, these tools may function as teaching aids and inform conduct of their review during its development.

#### Description

Systematic reviews that include randomized and/or non-randomized studies as evidence can be appraised with AMSTAR-2 and ROBIS. Other characteristics of AMSTAR-2 and ROBIS are summarized in Table 3.2. Both tools define categories for an overall rating; however, neither tool is intended to generate a total score by simply calculating the number of responses satisfying criteria for individual items.^4,6^ AMSTAR-2 focuses on the rigor of a review’s methods irrespective of the specific subject matter. ROBIS places emphasis on a review’s results section—this suggests it may be optimally applied by appraisers with some knowledge of the review’s topic as they may be better equipped to determine if certain procedures (or lack thereof) would impact the validity of a review’s findings.^98,100^ Reliability studies show AMSTAR-2 overall confidence ratings strongly correlate with the overall RoB ratings in ROBIS.^100,101^

**Table 3.2:** Comparison of AMSTAR-2 and ROBIS

Characteristic	AMSTAR-2	ROBIS
**Access**	https://amstar.ca/Amstar-2.php	http://www.bristol.ac.uk/population-health-sciences/projects/robis/robis-tool/
**User guidance**	Extensive	Extensive
**Review type applicability**	Intervention	Intervention, diagnostic, etiology, prognostic^**a**^
**Number of domains**	7 critical, 9 non-critical	4
**Items**		
**Total number**	16	29
**Response options**	Items # 1, 3, 5, 6, 10, 13, 14, 16: rated *yes* or *no* Items # 2, 4, 7, 8, 9^b^: rated *yes, partial yes*, or *no* Items # 11^b^; 12, 15: rated *yes, partial yes, no*, or *no meta-analysis*	24 assessment items: rated *yes, probably yes, no information, probably no*, and *no* 5 items regarding level of concern: rated *low, high, unclear*
**Overall rating**		
**Construct**	Confidence based on weaknesses in critical domains	Level of concern for risk of bias
**Categories**	High, moderate, low, and critically low	Low, high, unclear

^a^ ROBIS includes an optional first phase to assess the applicability of the review to the research question of interest. The tool may be applicable to other review types in addition to the four specified, although modification of this initial phase will be needed (personal communication via email, Penny Whiting, 28 Jan 2022).

^b^ Items #9 and #11 require separate responses for RCTs and NRSI.

Interrater reliability has been shown to be acceptable for AMSTAR-2^6,11,102^ and ROBIS^4,98,103^ but neither tool has been shown to be superior in this regard.^100,101,104,105^ Overall, variability in reliability for both tools has been reported across items, between pairs of raters, and between centers.^6,100,101,104^ The effects of appraiser experience on the results of AMSTAR-2 and ROBIS requires, further evaluation.^101,105^ Updates to both tools should address items shown to be prone to individual appraisers’ subjective biases and opinions^11,100^; this may involve modifications of the current domains and signaling questions as well as incorporation of methods to make an appraiser’s judgments more explicit. Future revisions of these tools may also consider the addition of standards for aspects of systematic review development currently lacking (eg, rating overall certainty of evidence,^99^ methods for synthesis without meta-analysis^105^) and removal of items that assess aspects of reporting that are thoroughly evaluated by PRISMA 2020.

#### Application

A good understanding of what is required to satisfy the standards of AMSTAR-2 and ROBIS involves study of the accompanying guidance documents written by the tools’ developers; these contain detailed descriptions of each item’s standards. In addition, accurate appraisal of a systematic review with either tool requires training. Most experts recommend independent assessment by at least two appraisers, with a process for resolving discrepancies as well as procedures to establish interrater reliability, such as pilot testing, a calibration phase or exercise, and development of predefined decision rules.^35,99–101,103,104,106^ These methods may, to some extent, address the challenges associated with the diversity in methodological training, subject matter expertise, and experience using the tools that are likely to exist among appraisers.

#### Uptake

The standards of AMSTAR, AMSTAR-2, and ROBIS have been used in many methodological studies and epidemiological investigations. However, the increased publication of overviews or umbrella reviews and CPGs has likely been a greater influence on the widening acceptance of these tools. Critical appraisal of the secondary studies considered evidence is essential to the trustworthiness of both the recommendations of CPGs and the conclusions of overviews. Currently both Cochrane^55^ and JBI^107^ recommend AMSTAR-2 and ROBIS in their guidance for authors of overviews or umbrella reviews. However, ROBIS and AMSTAR-2 were released in 2016 and 2017, respectively; thus, to date, limited data have been reported about the uptake of these tools or which of the two may be preferred.^21,106^ Currently, in relation to CPGs, AMSTAR-2 appears to be overwhelmingly popular compared to ROBIS. A Google Scholar search of this topic (search terms “AMSTAR 2 AND clinical practice guidelines,” “ROBIS AND clinical practice guidelines” 13 May 2022) found 12,700 hits for AMSTAR-2 and 1280 for ROBIS. The apparent greater appeal of AMSTAR-2 may relate to its longer track record given the original version of the tool was in use for 10 years prior to its update in 2017.

Barriers to the uptake of AMSTAR-2 and ROBIS include the real or perceived time and resources necessary to complete the items they include and appraisers’ confidence in their own ratings.^104^ Reports from comparative studies available to date indicate that appraisers find AMSTAR-2 questions, responses, and guidance to be clearer and simpler compared with ROBIS.^11,101,104,105^ This suggests that for appraisal of intervention systematic reviews, AMSTAR-2 may be a more practical tool than ROBIS, especially for novice appraisers.^101,103–105^ The unique characteristics of each tool, as well as their potential advantages and disadvantages, should be taken into consideration when deciding which tool should be used for an appraisal of a systematic review. In addition, the choice of one or the other may depend on how the results of an appraisal will be used; for example, a peer reviewer’s appraisal of a single manuscript versus an appraisal of multiple systematic reviews in an overview or umbrella review, CPG, or systematic methodological study.

Authors of overviews and CPGs report results of AMSTAR-2 and ROBIS appraisals for each of the systematic reviews they include as evidence. Ideally, an independent judgment of their appraisals can be made by the end users of overviews and CPGs; however, most stakeholders, including clinicians, are unlikely to have a sophisticated understanding of these tools. Nevertheless, they should at least be aware that AMSTAR-2 and ROBIS ratings reported in overviews and CPGs may be inaccurate because the tools are not applied as intended by their developers. This can result from inadequate training of the overview or CPG authors who perform the appraisals, or to modifications of the appraisal tools imposed by them. The potential variability in overall confidence and RoB ratings highlights why appraisers applying these tools need to support their judgments with explicit documentation; this allows readers to judge for themselves whether they agree with the criteria used by appraisers.^4,108^ When these judgments are explicit, the underlying rationale used when applying these tools can be assessed.^109^

#### Impact

Theoretically, we would expect an association of AMSTAR-2 with improved methodological rigor and an association of ROBIS with lower RoB in recent systematic reviews compared to those published before 2017. To our knowledge, this has not yet been demonstrated; however, like reports about the actual uptake of these tools, time will tell. Additional data on user experience is also needed to further elucidate the practical challenges and methodological nuances encountered with the application of these tools. This information could potentially inform the creation of unifying criteria to guide and standardize the appraisal of evidence syntheses.^109^

### Evaluation of reporting

Complete reporting is essential for users to establish the trustworthiness and applicability of a systematic review’s findings. Efforts to standardize and improve the reporting of systematic reviews resulted in the 2009 publication of the PRISMA statement^92^ with its accompanying explanation and elaboration document.^110^ This guideline was designed to help authors prepare a complete and transparent report of their systematic review. In addition, adherence to PRISMA is often used to evaluate the thoroughness of reporting of published systematic reviews.^111^ The updated version, PRISMA 2020,^93^ and its guidance document^112^ were published in 2021. Items on the original and updated versions of PRISMA are organized by the six basic review components they address (title, abstract, introduction, methods, results, discussion). The PRISMA 2020 update is a considerably expanded version of the original; it includes standards and examples for the 27 original and 13 additional reporting items that capture methodological advances and may enhance the replicability of reviews.^113^

The original PRISMA statement fostered the development of various PRISMA extensions (Table 3.3). These include reporting guidance for scoping reviews and reviews of diagnostic test accuracy and for intervention reviews that report on the following: harms outcomes, equity issues, the effects of acupuncture, the results of network meta-analyses and analyses of individual participant data. Detailed reporting guidance for specific systematic review components (abstracts, protocols, literature searches) is also available.

**Table 3.3:** PRISMA extensions

Systematic review type or component addressed	Acronym	Year	Link
PRISMA for systematic reviews with a focus on health equity^114^	PRISMA-E	2012	http://prisma-statement.org/Extensions/Equity
Reporting systematic reviews in journal and conference abstracts^115^	PRISMA for Abstracts	2015; 2020^a^	http://prisma-statement.org/Extensions/Protocols
PRISMA for systematic review protocols^116^	PRISMA-P	2015	http://prisma-statement.org/Extensions/Protocols
PRISMA for Network Meta-Analyses^117^	PRISMA-NMA	2015	http://prisma-statement.org/Extensions/NetworkMetaAnalysis
PRISMA for Individual Participant Data^118^	PRISMA-IPD	2015	http://prisma-statement.org/Extensions/IndividualPatientData
PRISMA for reviews including harms outcomes^119^	PRISMA-Harms	2016	http://prisma-statement.org/Extensions/Harms
PRISMA for diagnostic test accuracy^120^	PRISMA-DTA	2018	http://prisma-statement.org/Extensions/DTA
PRISMA for scoping reviews^121^	PRISMA-ScR	2018	http://prisma-statement.org/Extensions/ScopingReviews
PRISMA for acupuncture^122^	PRISMA-A	2019	http://prisma-statement.org/Extensions/Acupuncture
PRISMA for reporting literature searches^123^	PRISMA-S	2021	http://prisma-statement.org/Extensions/Searching

PRISMA, Preferred Reporting Items for Systematic Reviews and Meta-Analyses

^a^ Note the abstract reporting checklist is now incorporated into PRISMA 2020.^93^

#### Uptake and impact

The 2009 PRISMA standards^92^ for reporting have been widely endorsed by authors, journals, and EBM-related organizations. We anticipate the same for PRISMA 2020^93^ given its co-publication in multiple high-impact journals. However, to date, there is a lack of strong evidence for an association between improved systematic review reporting and endorsement of PRISMA 2009 standards.^43,111^ Most journals require a PRISMA checklist accompany submissions of systematic review manuscripts. However, the accuracy of information presented on these self-reported checklists is not necessarily verified. It remains unclear which strategies (eg, authors’ self-report of checklists, peer reviewer checks) might improve adherence to the PRISMA reporting standards; in addition, the feasibility of any potentially effective strategies must be taken into consideration given the structure and limitations of current research and publication practices.^124^

### Pitfalls and limitations of PRISMA, AMSTAR-2, and ROBIS

Misunderstanding of the roles of these tools and their misapplication may be widespread problems. PRISMA 2020 is a reporting guideline that is most beneficial if consulted when developing a review as opposed to merely completing a checklist when submitting to a journal; at that point, the review is finished, with good or bad methodological choices. However, PRISMA checklists evaluate how completely an element of review conduct was reported, but do not evaluate the caliber of conduct or performance of a review. Thus, review authors and readers should not think that a rigorous systematic review can be produced by simply following the PRISMA 2020 guidelines. Similarly, it is important to recognize that AMSTAR-2 and ROBIS are tools to evaluate the conduct of a review but do not substitute for conceptual methodological guidance. In addition, they are not intended to be simple checklists. In fact, they have the potential for misuse or abuse if applied as such; for example, by calculating a total score to make a judgment about a review’s overall confidence or RoB. Proper selection of a response for the individual items on AMSTAR-2 and ROBIS requires training or at least reference to their accompanying guidance documents.

Not surprisingly, it has been shown that compliance with the PRISMA checklist is not necessarily associated with satisfying the standards of ROBIS.^125^ AMSTAR and ROBIS were not available when PRISMA 2009 was developed; however, they were considered in the development of PRISMA 2020.^113^ Therefore, future studies may show a positive relationship between fulfillment of PRISMA 2020 standards for reporting and meeting the standards of tools evaluating methodological quality and RoB.

### Recommendations

Choice of an appropriate tool for the evaluation of a systematic review first involves identification of the underlying construct to be assessed. For systematic reviews of interventions, recommended tools include AMSTAR-2 and ROBIS for appraisal of conduct and PRISMA 2020 for completeness of reporting. All three tools were developed rigorously and provide easily accessible and detailed user guidance, which is necessary for their proper application and interpretation. When considering a manuscript for publication, training in these tools can sensitize peer reviewers and editors to major issues that may affect the review’s trustworthiness and completeness of reporting. Judgment of the overall certainty of a body of evidence and formulation of recommendations rely, in part, on AMSTAR-2 or ROBIS appraisals of systematic reviews. Therefore, training on the application of these tools is essential for authors of overviews and developers of CPGs. Peer reviewers and editors considering an overview or CPG for publication must hold their authors to a high standard of transparency regarding both the conduct and reporting of these appraisals.

## Part 4. Meeting conduct standards

Many authors, peer reviewers, and editors erroneously equate fulfillment of the items on the PRISMA checklist with superior methodological rigor. For direction on methodology, we refer them to available resources that provide comprehensive conceptual guidance^59,69^ as well as primers with basic step-by-step instructions.^1,126,127^ This section is intended to complement study of such resources by facilitating use of AMSTAR-2 and ROBIS, tools specifically developed to evaluate methodological rigor of systematic reviews. These tools are widely accepted by methodologists; however, in the general medical literature, they are not uniformly selected for the critical appraisal of systematic reviews.^88,96^

To enable their uptake, Table 4.1 links review components to the corresponding appraisal tool items. Expectations of AMSTAR-2 and ROBIS are concisely stated, and reasoning provided.

**Table 4.1:** Systematic review components linked to appraisal with AMSTAR-2 and ROBIS^a^

Review component	Corresponding item(s)		Expectation of AMSTAR-2 and/or ROBIS	Reasoning
	AMSTAR-2^b^	ROBIS		
**Research question(s)**	**#1**	**PHASE I**	**Appropriate for type of review (see** **Table 2.1**).	**Promotes conceptual clarity (see** **Table 2.1**).
**Protocol**	#2*	**#1.1, 4.2**	**Follows PRISMA-P; registration confirms developed** **a priori** **; deviations are documented in protocol and explained in review.**	**Guides authors and reviewers, limits scope, prevents arbitrary decisions, fosters collaboration, and reduces research waste.**
**Justification for study design inclusion decisions**	**#3**	**#1.2, 1.4, 2.3, 2.4**	**Explain reasons for study designs included in review.**	**Excessive exclusions narrow the field of vision and may introduce bias or limit the potential usefulness of research available to assess. Reviews of interventions should rarely be limited at this stage.**
**Evidence search**	#4*	**#2.1-2.4**	**Systematic and comprehensive without restrictions.**	**Mitigates author and publications bias, promotes diversity of understanding.**
Methods for study selection	#5	#2.5	All three components must be done in duplicate, and methods fully described.	Helps to mitigate CoI and bias; also may improve accuracy.
Methods for data extraction	#6	#3.1		
Methods for RoB assessment	NA	#3.5		
**List of studies excluded at full text level**	#7*	**#4.1**	**Indicate reasons for exclusion.**	**Improves confidence all eligible studies are included.**
Study description	#8	#3.2	Research design features, components of research question (eg, PICO), setting, funding sources.	Allows readers to understand the individual studies in detail.
**Tool for RoB assessment**	#9*	**#3.4**	**Use of reliable and valid tools appropriate for study design features.**	**Tools chosen must assess specific sources of bias required by AMSTAR-2 or ROBIS.**
**RoB assessment results**	**#12 (if MA), 13**	**#4.6, 3.4**	**Interpreted and discussed.**	**Allows readers to understand the details of RoB issues, optimally by each outcome investigated.**
Sources of funding	#10	NA	Identified for all included studies.	Can reveal CoI or bias.
**Synthesis methods**	#11* (if MA), 13*, 14	**#4.1, 4.3, 4.4**	**Appropriate methods for quantitative data with or without meta-analysis, including identification and discussion of heterogeneity.**	**Strengthens the ability to obtain more reliable results and make sound inferences.**
Publication bias	#15*	#4.5	Explored, diagrammed, and discussed.	Publication and other selective reporting biases are major threats to the validity of systematic reviews.
Author CoI	#16	NA	Disclosed, with management strategies described.	If CoI is identified, management strategies must be described to ensure confidence in the review.

CoI, conflict of interest; MA, meta-analysis; NA, not addressed; PICO, participant, intervention, comparison, outcome; PRISMA-P, Preferred Reporting Items for Systematic Review and Meta-Analysis Protocols; RoB, risk of bias.

^a^ Components shown in bold are chosen for elaboration in Part 4 for one (or both) of two reasons: 1) the component has been identified as potentially problematic for systematic review authors; and/or 2) the component is evaluated by standards of an AMSTAR-2 “critical” domain.

^b^ Critical domains of AMSTAR-2 are indicated by *.

Issues involved in meeting the standards for seven review components (identified in bold in Table 4.1) are addressed in detail. These were chosen for elaboration for one (or both) of two reasons: 1) the component has been identified as potentially problematic for systematic review authors based on consistent reports of their frequent AMSTAR-2 or ROBIS deficiencies^9,11,15,88,128,129^; and/or 2) the review component is judged by standards of an AMSTAR-2 “critical” domain. These have the greatest implications for how a systematic review will be appraised: if standards for any one of these critical domains are not met, the review is rated as having “critically low confidence.”

### Research question

Specific and unambiguous research questions may have more value for reviews that deal with hypothesis testing. Mnemonics for the various elements of research questions are suggested by JBI and Cochrane (Table 2.1). These prompt authors to consider the specialized methods involved for developing different types of systematic reviews; however, while inclusion of the suggested elements makes a review compliant with a particular review’s methods, it does not necessarily make a research question appropriate. Table 4.2 lists acronyms that may aid in developing the research question. They include overlapping concepts of importance in this time of proliferating reviews of uncertain value.^130^ If these issues are not prospectively contemplated, systematic review authors may establish an overly broad scope or develop runaway scope, allowing them to stray from predefined choices relating to key comparisons and outcomes.

**Table 4.2:** Research question development

Acronym	Meaning
**FINER** ^a^	**F** feasible, **I** interesting, **N** novel, **E** ethical, and **R** relevant
**SMART** ^b^	**S** specific, **M** measurable, **A** attainable, **R** relevant, **T** timely
**TOPICS + M** ^c^	**T** time, **O** outcomes, **P** population, **I** intervention, **C** context, **S** study design, plus **M** (effect) moderators

^a^ Cummings SR, Browner WS, Hulley SB. Conceiving the research question and developing the study plan. In: Hulley SB, Cummings SR, Browner WS, editors. Designing clinical research: an epidemiological approach; 4th ed. Lippincott Williams & Wilkins; 2007. p. 14–22.

^b^ Doran, GT. There’s a S.M.A.R.T. way to write management’s goals and objectives. Manage Rev. 1981;70:35-6.

^c^ Johnson BT, Hennessy EA. Systematic reviews and meta-analyses in the health sciences: best practice methods for research syntheses. Soc Sci Med. 2019;233:237–51.

Once a research question is established, searching on registry sites and databases for existing systematic reviews addressing the same or a similar topic is necessary in order to avoid contributing to research waste.^131^ Repeating an existing systematic review must be justified, for example, if previous reviews are out of date or methodologically flawed. A full discussion on replication of intervention systematic reviews, including a consensus checklist, can be found in the work of Tugwell and colleagues.^84^

### Protocol

Protocol development is considered a core component of systematic reviews.^125,126,132^ Review protocols may allow researchers to plan and anticipate potential issues, assess validity of methods, prevent arbitrary decision-making, and minimize bias that can be introduced by the conduct of the review. Registration of a protocol that allows public access promotes transparency of the systematic review’s methods and processes and reduces the potential for duplication.^132^ Thinking early and carefully about all the steps of a systematic review is pragmatic and logical and may mitigate the influence of the authors’ prior knowledge of the evidence.^133^ In addition, the protocol stage is when the scope of the review can be carefully considered by authors, reviewers, and editors; this may help to avoid production of overly ambitious reviews that include excessive numbers of comparisons and outcomes or are undisciplined in their study selection.

An association with attainment of AMSTAR standards in systematic reviews with published prospective protocols has been reported.^134^ However, completeness of reporting does not seem to be different in reviews with a protocol compared to those without one.^135^ PRISMA-P^116^ and its accompanying elaboration and explanation document^136^ can be used to guide and assess the reporting of protocols. A final version of the review should fully describe any protocol deviations. Peer reviewers may compare the submitted manuscript with any available pre-registered protocol; this is required if AMSTAR-2 or ROBIS are used for critical appraisal.

There are multiple options for the recording of protocols (Table 4.3). Some journals will peer review and publish protocols. In addition, many online sites offer date-stamped and publicly accessible protocol registration. Some of these are exclusively for protocols of evidence syntheses; others are less restrictive and offer researchers the capacity for data storage, sharing, and other workflow features. These sites document protocol details to varying extents and have different requirements.^137^ The most popular site for systematic reviews, the International Prospective Register of Systematic Reviews (PROSPERO), for example, only registers reviews that report on an outcome with direct relevance to human health. The PROSPERO record documents protocols for all types of reviews except literature and scoping reviews. Of note, PROSPERO requires authors register their review protocols prior to any data extraction.^133,138^ The electronic records of most of these registry sites allow authors to update their protocols and facilitate transparent tracking of protocol changes, which are not unexpected during the progress of the review.^139^

**Table 4.3:** Options for protocol registration of evidence syntheses

**Journals** ^a^	
BMJ Open	https://bmjopen.bmj.com/pages/authors/#protocol
BioMed Central	https://systematicreviewsjournal.biomedcentral.com/submission-guidelines/preparing-your-manuscript/protocol
JMIR Research Protocols	https://support.jmir.org/hc/en-us
World Journal of Meta-analysis	https://www.wjgnet.com/2308-3840/index.htm
**Exclusive systematic review registration sites**	
Cochrane^b^	https://community.cochrane.org/review-production/production-resources/proposing-and-registering-new-cochrane-reviews
JBI^c^	https://jbi.global/systematic-review-register
PROSPERO^d^	https://www.crd.york.ac.uk/prospero/
Research Registry: Registry of Systematic Reviews/Meta-Analyses^d^	https://www.researchregistry.com/browse-the-registry#registryofsystematicreviewsmeta-analyses/
International Platform of Registered Systematic Review and Meta-analysis Protocols (INPLASY)^d^	https://inplasy.com/
**Nonspecific research registration sites**	
Center for Open Science^d^	https://www.cos.io/initiatives/prereg
Protocols.io^d^	https://www.protocols.io/
**Data repositories** ^e^	
Figshare	https://figshare.com/
Open Science Framework	https://osf.io/
Zenodo	https://zenodo.org

^a^ Authors are advised to contact their target journal regarding submission of systematic review protocols.

^b^ Registration is restricted to approved review projects.

^c^ The JBI registry lists review projects currently underway by JBI-affiliated entities. These records include a review’s title, primary author, research question, and PICO elements. JBI recommends that authors register eligible protocols with PROSPERO.

^d^ See Pieper and Rombey^137^ for detailed characteristics of these five registries.

^e^ See Pieper and Rombey^137^ for other systematic review data repository options.

### Study design inclusion

For most systematic reviews, broad inclusion of study designs is recommended.^126^ This may allow comparison of results between contrasting study design types.^126^ Certain study designs may be considered preferable depending on the type of review and nature of the research question. However, prevailing stereotypes about what each study design does best may not be accurate. For example, in systematic reviews of interventions, randomized designs are typically thought to answer highly specific questions while non-randomized designs often are expected to reveal greater information about harms or real-word evidence.^126,140,141^ This may be a false distinction; randomized trials may be pragmatic,^142^ they may offer important (and more unbiased) information on harms,^143^ and data from non-randomized trials may not necessarily be more real-world-oriented.^144^

Moreover, there may not be any available evidence reported by RCTs for certain research questions; in some cases, there may not be any RCTs or NRSI. When the available evidence is limited to case reports and case series, it is not possible to test hypotheses nor provide descriptive estimates or associations; however, a systematic review of these studies can still offer important insights.^81,145^ When authors anticipate that limited evidence of any kind may be available to inform their research questions, a scoping review can be considered. Alternatively, decisions regarding inclusion of indirect as opposed to direct evidence can be addressed during protocol development.^146^ Including indirect evidence at an early stage of intervention systematic review development allows authors to decide if such studies offer any additional and/or different understanding of treatment effects for their population or comparison of interest. Issues of indirectness of included studies are accounted for later in the process, during determination of the overall certainty of evidence (see Part 5 for details).

### Evidence search

Both AMSTAR-2 and ROBIS require systematic and comprehensive searches for evidence. This is essential for any systematic review. Both tools discourage search restrictions based on language and publication source. Given increasing globalism in health care, the practice of including English-only literature should be avoided.^126^ There are many examples in which language bias (different results in studies published in different languages) has been documented.^147,148^ This does not mean that all literature, in all languages, is equally trustworthy^148^; however, the only way to formally probe for the potential of such biases is to consider all languages in the initial search. The gray literature and a search of trials may also reveal important details about topics that would otherwise be missed.^149–151^ Again, inclusiveness will allow review authors to investigate whether results differ in gray literature and trials.^41,151–153^

Authors should make every attempt to complete their review within one year as that is the likely viable life of a search.^1^ If that is not possible, the search should be updated close to the time of completion.^154^ Different research topics may warrant less of a delay, for example, in rapidly changing fields (as in the case of the COVID-19 pandemic), even one month may radically change the available evidence.

### Excluded studies

AMSTAR-2 requires authors to provide references for any studies excluded at the full-text phase of study selection along with reasons for exclusion; this allows readers to feel confident that all relevant literature has been considered for inclusion and that exclusions are defensible.

### Risk of bias assessment of included studies

The design of the studies included in a systematic review (eg, RCT, cohort, case series) should not be equated with appraisal of its RoB. To meet AMSTAR-2 and ROBIS standards, systematic review authors must examine RoB issues specific to the design of each primary study they include as evidence. It is unlikely that a single RoB appraisal tool will be suitable for all research designs. In addition to tools for randomized and non-randomized studies, specific tools are available for evaluation of RoB in case reports and case series^82^ and single-case experimental designs.^155,156^ Note the RoB tools selected must meet the standards of the appraisal tool used to judge the conduct of the review. For example, AMSTAR-2 identifies four sources of bias specific to RCTs and NRSI that must be addressed by the RoB tool(s) chosen by the review authors. The Cochrane RoB-2^157^ tool for RCTs and ROBINS-I^158^ for NRSI for RoB assessment meet the AMSTAR-2 standards. Appraisers on the review team should not modify any RoB tool without complete transparency and acknowledgment that they have invalidated the interpretation of the tool as intended by its developers.^159^ Conduct of RoB assessments is not addressed in AMSTAR-2; to meet ROBIS standards, two independent reviewers should complete RoB assessments of included primary studies.

Implications of the RoB assessments must be explicitly discussed and considered in the conclusions of the review. Discussion of the overall RoB of included studies may consider the weight of the studies at high RoB, the importance of the sources of bias in the studies being summarized, and if their importance differs in relationship to the outcomes reported. If a meta-analysis is performed, serious concerns for RoB of individual studies should be accounted for in these results as well. If the results of the meta-analysis for a specific outcome change when studies at high RoB are excluded, readers will have a more accurate understanding of this body of evidence. However, while investigating the potential impact of specific biases is a useful exercise, it is important to avoid over-interpretation, especially when there are sparse data.

### Synthesis methods for quantitative data

Syntheses of quantitative data reported by primary studies are broadly categorized as one of two types: meta-analysis, and synthesis without meta-analysis (Table 4.4). Before deciding on one of these methods, authors should seek methodological advice about whether reported data can be transformed or used in other ways to provide a consistent effect measure across studies.^160,161^

**Table 4.4:** Common methods for quantitative synthesis

Methods	Statistical technique	Reported data	Presentation
**Meta-analysis** Aggregate data^a^ Individual participant data^c^	Weighted average of effect estimates	Pairwise comparisons of effect estimates, CI Overall effect estimate, CI, *P* value Evaluation of heterogeneity	Forest plot^b^ with summary statistic for average effect estimate
Network^a^	Variable^d^	The interventions, which are compared directly versus indirectly	Network diagram or graph, tabular presentations
		Comparisons of relative effects between any pair of interventions	Effect estimates for intervention pairings
		Summary relative effects for pair-wise comparisons with evaluations of inconsistency and heterogeneity	Forest plot, other methods
		Treatment rankings (ie, probability that an intervention is among the best options)	Rankogram plot
**Synthesis without meta-analysis** ^e^	Summarizing effect estimates from separate studies (without combination that would provide an average effect estimate)	Range and distribution of observed effects such as median, interquartile range, range	Box-and-whisker plot, bubble plot Forest plot (without summary effect estimate)
	Combining *P* values	Combined *P* value, number of studies	Albatross plot (study sample size against *P* values per outcome)
	Vote counting by direction of effect (eg, favors intervention over the comparator)	Proportion of studies with an effect in the direction of interest, CI, *P* value	Harvest plot, effect direction plot

CI, confidence interval (or credible interval, if analysis is done in Bayesian framework).

^a^ See text for descriptions of the types of data combined in each of these approaches.

^b^ See Supplementary File 4 for guidance on the structure and presentation of forest plots.

^c^ General approach is similar to aggregate data meta-analysis but there are substantial differences relating to data collection and checking and analysis.^162^ This approach to syntheses is applicable to intervention, diagnostic, and prognostic systematic reviews.^163^

^d^ Examples include meta-regression, hierarchical, and multivariate approaches.^164^

^e^ In-depth guidance and illustrations of these methods are provided in Chapter 12 of the Cochrane Handbook.^160^

#### Meta-analysis

Systematic reviews that employ meta-analysis should not be referred to simply as “meta-analyses.” The term meta-analysis strictly refers to a specific statistical technique used when study effect estimates and their variances are available, yielding a quantitative summary of results. In general, methods for meta-analysis involve use of a weighted average of effect estimates from two or more studies. If considered carefully, meta-analysis increases the precision of the estimated magnitude of effect and can offer useful insights about heterogeneity and estimates of effects. We refer to standard references for a thorough introduction and formal training.^165–167^

There are three common approaches to meta-analysis in current health care–related systematic reviews (Table 4.4). Aggregate meta-analyses is the most familiar to authors of evidence syntheses and their end users. This standard meta-analysis combines data on effect estimates reported by studies that investigate similar research questions involving direct comparisons of an intervention and comparator. Results of these analyses provide a single summary intervention effect estimate. If the included studies in a systematic review measure an outcome differently, their reported results may be transformed to make them comparable.^161^ Forest plots visually present essential information about the individual studies and the overall pooled analysis (see Supplemental File 4 for details: http://links.lww.com/SRX/A15).

Less familiar and more challenging meta-analytical approaches used in secondary research include individual participant data (IPD) and network meta-analyses (NMA); PRISMA extensions provide reporting guidelines for both.^117,118^ In IPD, the raw data on each participant from each eligible study are re-analyzed as opposed to the study-level data analyzed in aggregate data meta-analyses.^168^ This may offer advantages, including the potential for limiting concerns about bias and allowing more robust analyses.^163^ As suggested by the description on Table 4.4, NMA is a complex statistical approach. It combines aggregate data^169^ or IPD^170^ for effect estimates from direct and indirect comparisons reported in two or more studies of three or more interventions. This makes it a potentially powerful statistical tool; while multiple interventions are typically available to treat a condition, few have been evaluated in head-to-head trials.^171^ Both IPD and NMA facilitate a broader scope, and potentially provide more reliable and/or detailed results; however, compared to standard aggregate data meta-analyses, their methods are more complicated, time-consuming, and resource-intensive, and they have their own biases, so one needs sufficient funding, technical expertise, and preparation to employ them successfully.^41,172,173^

Several items in AMSTAR-2 and ROBIS address meta-analysis; thus, understanding the strengths, weaknesses, assumptions, and limitations of methods for meta-analyses is important. According to the standards of both tools, plans for a meta-analysis must be addressed in the review protocol, including reasoning, description of the type of quantitative data to be synthesized, and the methods planned for combining the data. This should not consist of stock statements describing conventional meta-analysis techniques; rather, authors are expected to anticipate issues specific to their research questions. Concern for the lack of training in meta-analysis methods among systematic review authors cannot be overstated. For those with training, the use of popular software (eg, RevMan,^174^ MetaXL,^175^ JBI SUMARI^176^) may facilitate exploration of these methods; however, such programs cannot substitute for the accurate interpretation of the results of meta-analyses, especially for more complex meta-analytical approaches.

#### Synthesis without meta-analysis

There are varied reasons a meta-analysis may not be appropriate or desirable.^160,161^ Syntheses that informally use statistical methods other than meta-analysis are variably referred to as descriptive, narrative, or qualitative syntheses or summaries; these terms are also applied to syntheses that make no attempt to statistically combine data from individual studies. However, use of such imprecise terminology is discouraged; in order to fully explore the results of any type of synthesis, some narration or description is needed to supplement the data visually presented in tabular or graphic forms.^63,177^ In addition, the term “qualitative synthesis” is easily confused with a synthesis of qualitative data in a qualitative or mixed methods review. “Syntheses without meta-analysis” is currently the preferred description of other ways to combine quantitative data from two or more studies. Use of this specific terminology when referring to these types of syntheses also implies the application of formal methods (Table 4.4).

Methods for syntheses without meta-analysis involve structured presentations of the data in any tables and plots. In comparison to narrative descriptions of each study, these are designed to more effectively and transparently show patterns and convey detailed information about the data; they also allow informal exploration of heterogeneity.^178^ In addition, acceptable quantitative statistical methods (Table 4.4) are formally applied; however, it is important to recognize these methods have significant limitations for the interpretation of the effectiveness of an intervention.^160^ Nevertheless, when meta-analysis is not possible, the application of these methods is less prone to bias compared with an unstructured narrative description of included studies.^178,179^

Vote counting is commonly used in systematic reviews and involves a tally of studies reporting results that meet some threshold of importance applied by review authors. Until recently, it has not typically been identified as a method for synthesis without meta-analysis. Guidance on an acceptable vote counting method based on direction of effect is currently available^160^ and should be used instead of narrative descriptions of such results (eg, “more than half the studies showed improvement”; “only a few studies reported adverse effects”; “7 out of 10 studies favored the intervention”). Unacceptable methods include vote counting by statistical significance or magnitude of effect or some subjective rule applied by the authors.

AMSTAR-2 and ROBIS standards do not explicitly address conduct of syntheses without meta-analysis, although AMSTAR-2 items 13 and 14 might be considered relevant. Guidance for the complete reporting of syntheses without meta-analysis for systematic reviews of interventions is available in the Synthesis without Meta-analysis (SWiM) guideline^180^ and methodological guidance is available in the Cochrane Handbook.^160,181^

### Recommendations

Familiarity with AMSTAR-2 and ROBIS makes sense for authors of systematic reviews as these appraisal tools will be used to judge their work; however, training is necessary for authors to truly appreciate and apply methodological rigor. Moreover, judgment of the potential contribution of a systematic review to the current knowledge base goes beyond meeting the standards of AMSTAR-2 and ROBIS. These tools do not explicitly address some crucial concepts involved in the development of a systematic review; this further emphasizes the need for author training.

We recommend that systematic review authors incorporate specific practices or exercises when formulating a research question at the protocol stage, These should be designed to raise the review team’s awareness of how to prevent research and resource waste^84,130^ and to stimulate careful contemplation of the scope of the review.^30^ Authors’ training should also focus on justifiably choosing a formal method for the synthesis of quantitative and/or qualitative data from primary research; both types of data require specific expertise. For typical reviews that involve syntheses of quantitative data, statistical expertise is necessary, initially for decisions about appropriate methods,^160,161^ and then to inform any meta-analyses^167^ or other statistical methods applied.^160^

## Part 5. Rating overall certainty of evidence

Report of an overall certainty of evidence assessment in a systematic review is an important new reporting standard of the updated PRISMA 2020 guidelines.^93^ Systematic review authors are well acquainted with assessing RoB in individual primary studies, but much less familiar with assessment of overall certainty across an entire body of evidence. Yet a reliable way to evaluate this broader concept is now recognized as a vital part of interpreting the evidence.

### Background

Historical systems for rating evidence are based on study design and usually involve hierarchical levels or classes of evidence that use numbers and/or letters to designate the level/class. These systems were endorsed by various EBM-related organizations. Professional societies and regulatory groups then widely adopted them, often with modifications for application to the available primary research base in specific clinical areas. In 2002, a report issued by the AHRQ identified 40 systems to rate quality of a body of evidence.^182^ A critical appraisal of systems used by prominent health care organizations published in 2004 revealed limitations in sensibility, reproducibility, applicability to different questions, and usability to different end users.^183^ Persistent use of hierarchical rating schemes to describe overall quality continues to complicate the interpretation of evidence. This is indicated by recent reports of poor interpretability of systematic review results by readers^184–186^ and misleading interpretations of the evidence related to the “spin” systematic review authors may put on their conclusions.^50,187^

Recognition of the shortcomings of hierarchical rating systems raised concerns that misleading clinical recommendations could result even if based on a rigorous systematic review. In addition, the number and variability of these systems were considered obstacles to quick and accurate interpretations of the evidence by clinicians, patients, and policymakers.^183^ These issues contributed to the development of the GRADE approach. An international working group, that continues to actively evaluate and refine it, first introduced GRADE in 2004.^188^ Currently more than 110 organizations from 19 countries around the world have endorsed or are using GRADE.^189^

### GRADE approach to rating overall certainty

GRADE offers a consistent and sensible approach for two separate processes: rating the overall certainty of a body of evidence and the strength of recommendations. The former is the expected conclusion of a systematic review, while the latter is pertinent to the development of CPGs. As such, GRADE provides a mechanism to bridge the gap from evidence synthesis to application of the evidence for informed clinical decision-making.^27,190^ We briefly examine the GRADE approach but only as it applies to rating overall certainty of evidence in systematic reviews.

In GRADE, use of “certainty” of a body of evidence is preferred over the term “quality.”^191^ Certainty refers to the level of confidence systematic review authors have that, for each outcome, an effect estimate represents the true effect. The GRADE approach to rating confidence in estimates begins with identifying the study type (RCT or NRSI) and then systematically considers criteria to rate the certainty of evidence up or down (Table 5.1).

**Table 5.1:** GRADE criteria for rating certainty of evidence

Reasons for rating down^a^	Reasons for rating up^195^^,^^b^
Risk of bias^196^	Large magnitude of effect
Imprecision^197^	Dose-response gradient
Inconsistency^198^	All residual confounding would decrease magnitude of effect (in situations with an effect)
Indirectness^199^	
Publication bias^200^	

^a^ Applies to randomized studies.

^b^ Applies to non-randomized studies.

This process results in assignment of one of the four GRADE certainty ratings to each outcome; these are clearly conveyed with the use of basic interpretation symbols (Table 5.2).^192^ Notably, when multiple outcomes are reported in a systematic review, each outcome is assigned a unique certainty rating; thus different levels of certainty may exist in the body of evidence being examined.

**Table 5.2:** GRADE certainty ratings and their interpretation symbols^a^

⊕⊕⊕⊕ High: We are very confident that the true effect lies close to that of the estimate of the effect.
⊕⊕⊕○ Moderate: We are moderately confident in the effect estimate: the true effect is likely to be close to the estimate of the effect, but there is a possibility that it is substantially different.
⊕⊕○○ Low: Our confidence in the effect estimate is limited: the true effect may be substantially different from the estimate of the effect.
⊕○○○ Very low: We have very little confidence in the effect estimate: the true effect is likely to be substantially different from the estimate of effect.

^a^ Adapted from the GRADE Handbook^192^

GRADE’s developers acknowledge some subjectivity is involved in this process.^193^ In addition, they emphasize that both the criteria for rating evidence up and down (Table 5.1) as well as the four overall certainty ratings (Table 5.2) reflect a continuum as opposed to discrete categories.^194^ Consequently, deciding whether a study falls above or below the threshold for rating up or down may not be straightforward, and preliminary overall certainty ratings may be intermediate (eg, between low and moderate). Thus, the proper application of GRADE requires systematic review authors to take an overall view of the body of evidence and explicitly describe the rationale for their final ratings.

### Advantages of GRADE

Outcomes important to the individuals who experience the problem of interest maintain a prominent role throughout the GRADE process.^191^ These outcomes must inform the research questions (eg, PICO [population, intervention, comparator, outcome]) that are specified a priori in a systematic review protocol. Evidence for these outcomes is then investigated and each critical or important outcome is ultimately assigned a certainty of evidence as the end point of the review. Notably, limitations of the included studies have an impact at the outcome level. Ultimately, the certainty ratings for each outcome reported in a systematic review are considered by guideline panels. They use a different process to formulate recommendations that involves assessment of the evidence across outcomes.^201^ It is beyond our scope to describe the GRADE process for formulating recommendations; however, it is critical to understand how these 2 outcome-centric concepts of certainty of evidence in the GRADE framework are related and distinguished. An in-depth illustration using examples from recently published evidence syntheses and CPGs is provided in Supplemental File 5A (Table SF5A-1): http://links.lww.com/SRX/A16.

The GRADE approach is applicable irrespective of whether the certainty of the primary research evidence is high or very low; in some circumstances, indirect evidence of higher certainty may be considered if direct evidence is unavailable or of low certainty.^27^ In fact, most interventions and outcomes in medicine have low or very low certainty of evidence based on GRADE and there seems to be no major improvement over time.^202,203^ This is still a very important (even if sobering) realization for calibrating our understanding of medical evidence. A major appeal of the GRADE approach is that it offers a common framework that enables authors of evidence syntheses to make complex judgments about evidence certainty and to convey these with unambiguous terminology. This prevents some common mistakes made by review authors, including overstating results (or under-reporting harms)^187^ and making recommendations for treatment. This is illustrated in Table SF5A-2 (Supplemental File 5A: http://links.lww.com/SRX/A16), which compares the concluding statements made about overall certainty in a systematic review with and without application of the GRADE approach.

Theoretically, application of GRADE should improve consistency of judgments about certainty of evidence, both between authors and across systematic reviews. In one empirical evaluation conducted by the GRADE Working Group, interrater reliability of two individual raters assessing certainty of the evidence for a specific outcome increased from ~0.3 without using GRADE to ~0.7 by using GRADE.^204^ However, others report variable agreement among those experienced in GRADE assessments of evidence certainty.^190^ Like any other tool, GRADE requires training in order to be properly applied. The intricacies of the GRADE approach and the necessary subjectivity involved suggest that improving agreement may require strict rules for its application; alternatively, use of general guidance and consensus among review authors may result in less consistency but provide important information for the end user.^190^

### GRADE caveats

Simply invoking “the GRADE approach” does not automatically ensure GRADE methods were employed by authors of a systematic review (or developers of a CPG). Table 5.3 lists the criteria the GRADE Working Group has established for this purpose. These criteria highlight the specific terminology and methods that apply to rating the certainty of evidence for outcomes reported in a systematic review,^191^ which is different from rating overall certainty across outcomes considered in the formulation of recommendations.^205^ Modifications of standard GRADE methods and terminology are discouraged as these may detract from GRADE’s objectives to minimize conceptual confusion and maximize clear communication.^206^

**Table 5.3:** Criteria for using GRADE in a systematic review^a^

1. The certainty in the evidence (also known as quality of evidence or confidence in the estimates) should be defined consistently with the definitions used by the GRADE Working Group.
2. Explicit consideration should be given to each of the GRADE domains for assessing the certainty in the evidence (although different terminology may be used).
3. The overall certainty in the evidence should be assessed for each important outcome using four or three categories (such as high, moderate, low, and/or very low) and definitions for each category that are consistent with the definitions used by the GRADE Working Group.
4. Evidence summaries … should be used as the basis for judgments about the certainty in the evidence.

^a^ Adapted from the GRADE Working Group^206^; this list does not contain the additional criteria that apply to the development of a clinical practice guideline.

Nevertheless, GRADE is prone to misapplications,^207,208^ which can distort a systematic review’s conclusions about the certainty of evidence. Systematic review authors without proper GRADE training are likely to misinterpret the terms “quality” and “grade” and to misunderstand the constructs assessed by GRADE versus other appraisal tools. For example, review authors may reference the standard GRADE certainty ratings (Table 5.2) to describe evidence for their outcome(s) of interest. However, these ratings are invalidated if authors omit or inadequately perform RoB evaluations of each included primary study. Such deficiencies in RoB assessments are unacceptable but not uncommon, as reported in methodological studies of systematic reviews and overviews.^104,186,209,210^ GRADE ratings are also invalidated if review authors do not formally address and report on the other criteria (Table 5.1) necessary for a GRADE certainty rating.

Other caveats pertain to application of a GRADE certainty of evidence rating in various types of evidence syntheses. Current adaptations of GRADE are described in Supplemental File 5B (http://links.lww.com/SRX/A17) and included on Table 6.3, which is introduced in the next section.

### Recommendations

The expected culmination of a systematic review should be a rating of overall certainty of a body of evidence for each outcome reported. The GRADE approach is recommended for making these judgments for outcomes reported in systematic reviews of interventions and can be adapted for other types of reviews. This represents the initial step in the process of making recommendations based on evidence syntheses. Peer reviewers should ensure authors meet the minimal criteria for supporting the GRADE approach when reviewing any evidence synthesis that reports certainty ratings derived using GRADE. Authors and peer reviewers of evidence syntheses unfamiliar with GRADE are encouraged to seek formal training and take advantage of the resources available on the GRADE website.^211,212^

## Part 6. Concise Guide to best practices

Accumulating data in recent years suggest that many evidence syntheses (with or without meta-analysis) are not reliable. This relates in part to the fact that their authors, who are often clinicians, can be overwhelmed by the plethora of ways to evaluate evidence. They tend to resort to familiar but often inadequate, inappropriate, or obsolete methods and tools and, as a result, produce unreliable reviews. These manuscripts may not be recognized as such by peer reviewers and journal editors who may disregard current standards. When such a systematic review is published or included in a CPG, clinicians and stakeholders tend to believe that it is trustworthy. A vicious cycle in which inadequate methodology is rewarded and potentially misleading conclusions are accepted is thus supported. There is no quick or easy way to break this cycle; however, increasing awareness of best practices among all these stakeholder groups, who often have minimal (if any) training in methodology, may begin to mitigate it. This is the rationale for inclusion of Parts 2 through 5 in this guidance document. These sections present core concepts and important methodological developments that inform current standards and recommendations. We conclude by taking a direct and practical approach.

Inconsistent and imprecise terminology used in the context of development and evaluation of evidence syntheses is problematic for authors, peer reviewers and editors, and may lead to the application of inappropriate methods and tools. In response, we endorse use of the basic terms (Table 6.1) defined in the PRISMA 2020 statement.^93^ In addition, we have identified several problematic expressions and nomenclature. In Table 6.2, we compile suggestions for preferred terms less likely to be misinterpreted.

**Table 6.1:** Terms relevant to the reporting of health care–related evidence syntheses^a^

**Systematic review:** A review that uses explicit, systematic methods to collate and synthesize findings of studies that address a clearly formulated question.
**Statistical synthesis:** The combination of quantitative results of two or more studies. This encompasses meta-analysis of effect estimates and other methods, such as combining *P* values, calculating the range and distribution of observed effects, and vote counting based on the direction of effect.
**Meta-analysis of effect estimates:** A statistical technique used to synthesize results when study effect estimates and their variances are available, yielding a quantitative summary of results.
**Outcome**: An event or measurement collected for participants in a study (such as quality of life, mortality).
**Result**: The combination of a point estimate (such as a mean difference, risk ratio or proportion) and a measure of its precision (such as a confidence/credible interval) for a particular outcome.
**Report:** A document (paper or electronic) supplying information about a particular study. It could be a journal article, preprint, conference abstract, study register entry, clinical study report, dissertation, unpublished manuscript, government report, or any other document providing relevant information.
**Record**: The title or abstract (or both) of a report indexed in a database or website (such as a title or abstract for an article indexed in MEDLINE). Records that refer to the same report (such as the same journal article) are “duplicates”; however, records that refer to reports that are merely similar (such as a similar abstract submitted to two different conferences) should be considered unique.
**Study**: An investigation, such as a clinical trial, that includes a defined group of participants and one or more interventions and outcomes. A “study” might have multiple reports. For example, reports could include the protocol, statistical analysis plan, baseline characteristics, results for the primary outcome, results for harms, results for secondary outcomes, and results for additional mediator and moderator analyses.

^a^ From Page and colleagues^93^

**Table 6.2:** Terminology suggestions for health care–related evidence syntheses.

Preferred	Potentially problematic
Evidence synthesis with meta-analysis Systematic review with meta-analysis	Meta-analysis
Overview or umbrella review	Systematic review of systematic reviews Review of reviews Meta-review
Randomized	Experimental
Non-randomized	Observational
Single case experimental design	Single-subject research N-of-1 design
Case report or case series	Descriptive study
Methodological quality	Quality
Certainty of evidence	Quality of evidence Grade of evidence Level of evidence Strength of evidence
Qualitative systematic review	Qualitative synthesis
Synthesis of qualitative data^a^	Qualitative synthesis
Synthesis without meta-analysis	Narrative synthesis^b^, narrative summary Qualitative synthesis Descriptive synthesis, descriptive summary

^a^ For example, meta-aggregation, meta-ethnography, critical interpretative synthesis, realist synthesis.

^b^ This term may best apply to the synthesis in a mixed methods systematic review in which data from different types of evidence (eg, qualitative, quantitative, economic) are summarized.^64^

We also propose a Concise Guide (Table 6.3) that summarizes the methods and tools recommended for the development and evaluation of nine types of evidence syntheses. Suggestions for specific tools are based on the rigor of their development as well as the availability of detailed guidance from their developers to ensure their proper application. The formatting of the Concise Guide addresses a well-known source of confusion by clearly distinguishing the underlying methodological constructs that these tools were designed to assess. Important clarifications and explanations follow in the guide’s footnotes; associated websites, if available, are listed in Supplemental File 6: http://links.lww.com/SRX/A18.

**Table 6.3:** Concise guide to best practices for evidence syntheses, version 1.0^a^

Resources	Intervention	Diagnostic	Prognostic	Qualitative or mixed methods	Prevalence and incidence	Etiology and risk	Measurement properties	Overviews (umbrella reviews)	Scoping reviews
**Methodological guidance**	Cochrane^b^, JBI	Cochrane, JBI	Cochrane	Cochrane, JBI	JBI	JBI	JBI	Cochrane, JBI	JBI
**Reporting** ^**c**^ Protocol Systematic review Synthesis without meta-analysis	PRISMA-P^116^	PRISMA-P	PRISMA-P	PRISMA-P	PRISMA-P	PRISMA-P	PRISMA-P	PRISMA-P	PRISMA-P
	PRISMA 2020^112^	PRISMA-DTA^120^	PRISMA 2020	eMERGe^213^^,^ ^d^ ENTREQ^214^^,^ ^d^	PRISMA 2020	PRISMA 2020	PRISMA 2020	PRIOR^215^	PRISMA-ScR^121^
	SWiM^180^		SWiM^e^		SWiM^e^	SWiM^e^	SWiM^e^		
**RoB assessment of included studies** ^f^	For RCTs: Cochrane RoB2^157^ For NRSI: ROBINS-I^158^ Other primary research^g^	QUADAS-2^216^	Factor review QUIPS^217^ Model review PROBAST^65^	CASP qualitative checklist^218^ JBI critical appraisal checklist^219^^,^ ^h^	JBI checklist for studies reporting prevalence data^220^	For NRSI: ROBINS-I^158^ Other primary research^g^	COSMIN RoB Checklist^67^	AMSTAR-2^6^ or ROBIS^4^	Not required^i^
**Overall level of evidence certainty**	GRADE^27^	GRADE adaptation^j^	GRADE adaptation^k^	CERQual^221^ ConQual^222^^,^ ^l^	GRADE adaptation^m^	Risk factors^n^	GRADE adaptation^o^	GRADE (for intervention reviews) Risk factors^n^	Not applicable

AMSTAR, A MeaSurement Tool to Assess Systematic Reviews; CASP, Critical Appraisal Skills Programme; CERQual, Confidence in the Evidence from Reviews of Qualitative research; ConQual, Establishing Confidence in the output of Qualitative research synthesis; COSMIN, COnsensus-based Standards for the selection of health Measurement Instruments; DTA, diagnostic test accuracy; eMERGe, meta-ethnography reporting guidance; ENTREQ, enhancing transparency in reporting the synthesis of qualitative research; GRADE, Grading of Recommendations Assessment, Development and Evaluation; NRSI, non-randomized studies of interventions; P, protocol; PRIOR, Preferred Reporting Items for Overviews of Reviews; PRISMA, Preferred Reporting Items for Systematic Reviews and Meta-Analyses; PROBAST, Prediction model Risk Of Bias ASsessment Tool; QUADAS, quality assessment of studies of diagnostic accuracy included in systematic reviews; QUIPS, Quality In Prognosis Studies; RCT, randomized controlled trial; RoB, risk of bias; ROBINS-I, Risk Of Bias In Non-randomised Studies of Interventions; ROBIS, Risk of Bias in Systematic Reviews; ScR, scoping review; SWiM, systematic review without meta-analysis.

^a^ Superscript numbers represent citations provided in the main reference list. Supplemental File 6 lists links to available online resources for the methods and tools included in the Concise Guide.

^b^ The MECIR manual^30^ provides Cochrane’s specific standards for both reporting and conduct of intervention systematic reviews and protocols.

^c^ Editorial and peer reviewers can evaluate completeness of reporting in submitted manuscripts using these tools. Authors may be required to submit a self-reported checklist for the applicable tools.

^d^ The decision flowchart described by Flemming and colleagues^223^ is recommended for guidance on how to choose the best approach to reporting for qualitative reviews.

^e^ SWiM was developed for intervention studies reporting quantitative data. However, if there is not a more directly relevant reporting guideline, SWiM may prompt reviewers to consider the important details to report. (Personal Communication via email, Mhairi Campbell, 14 Dec 2022).

^f^ JBI recommends their own tools for the critical appraisal of various quantitative primary study designs included in systematic reviews of intervention effectiveness, prevalence and incidence, and etiology and risk as well as for the critical appraisal of systematic reviews included in umbrella reviews. However, except for the JBI checklists for studies reporting prevalence data and qualitative research, the development, validity, and reliability of these tools are not well documented.

^g^ Studies that are not RCTs or NRSI require tools developed specifically to evaluate their design features. Examples include single case experimental design^155,156^ and case reports and series.^82^

^h^ The evaluation of methodological quality of studies included in a synthesis of qualitative research is debatable.^224^ Authors may select a tool appropriate for the type of qualitative synthesis methodology employed. The CASP Qualitative Checklist^218^ is an example of a published, commonly used tool that focuses on assessment of the methodological strengths and limitations of qualitative studies. The JBI Critical Appraisal Checklist for Qualitative Research^219^ is recommended for reviews using a meta-aggregative approach.

^i^ Consider including risk of bias assessment of included studies if this information is relevant to the research question; however, scoping reviews do not include an assessment of the overall certainty of a body of evidence.

^j^ Guidance available from the GRADE Working Group^225,226^; also recommend consultation with the Cochrane diagnostic methods group.

^k^ Guidance available from the GRADE Working Group^227^; also recommend consultation with Cochrane prognostic methods group.

^l^ Used for syntheses in reviews with a meta-aggregative approach.^224^

^m^ Chapter 5 in the *JBI Manual for Evidence Synthesis* offers guidance on how to adapt GRADE to prevalence and incidence reviews.^69^

^n^ Janiaud and colleagues suggest criteria for evaluating evidence certainty for meta-analyses of non-randomized studies evaluating risk factors.^228^

^o^ The COSMIN user manual provides details on how to apply GRADE in systematic reviews of measurement properties.^229^

To encourage uptake of best practices, journal editors may consider adopting or adapting the Concise Guide in their instructions to authors and peer reviewers of evidence syntheses. Given the evolving nature of evidence synthesis methodology, the suggested methods and tools are likely to require regular updates. Authors of evidence syntheses should monitor the literature to ensure they are employing current methods and tools. Some types of evidence syntheses (eg, rapid, economic, methodological) are not included in the Concise Guide; for these, authors are advised to obtain recommendations for acceptable methods by consulting with their target journal.

## Conclusion

We encourage the appropriate and informed use of the methods and tools discussed throughout this commentary and summarized in the Concise Guide (Table 6.3). However, we caution against their application in a perfunctory or superficial fashion. This is a common pitfall among authors of evidence syntheses, especially as the standards of such tools become associated with acceptance of a manuscript by a journal. Consequently, published evidence syntheses may show improved adherence to the requirements of these tools without necessarily making genuine improvements in their performance.

In line with our main objective, the suggested tools in the Concise Guide address the reliability of evidence syntheses; however, we recognize that the utility of systematic reviews is an equally important concern. An unbiased and thoroughly reported evidence synthesis may still not be highly informative if the evidence itself that is summarized is sparse, weak and/or biased.^24^ Many intervention systematic reviews, including those developed by Cochrane^203^ and those applying GRADE,^202^ ultimately find no evidence, or find the evidence to be inconclusive (eg, “weak,” “mixed,” or of “low certainty”). This often reflects the primary research base; however, it is important to know what is known (or not known) about a topic when considering an intervention for patients and discussing treatment options with them.

Alternatively, the frequency of “empty” and inconclusive reviews published in the medical literature may relate to limitations of conventional methods that focus on hypothesis testing; these have emphasized the importance of statistical significance in primary research and effect sizes from aggregate meta-analyses.^183^ It is becoming increasingly apparent that this approach may not be appropriate for all topics.^130^ Development of the GRADE approach has facilitated a better understanding of significant factors (beyond effect size) that contribute to the overall certainty of evidence. Other notable responses include the development of integrative synthesis methods for the evaluation of complex interventions,^230,231^ the incorporation of crowdsourcing and machine learning into systematic review workflows (eg the Cochrane Evidence Pipeline),^2^ the shift in paradigm to living systemic review and NMA platforms,^232,233^ and the proposal of a new evidence ecosystem that fosters bidirectional collaborations and interactions among a global network of evidence synthesis stakeholders.^234^ These evolutions in data sources and methods may ultimately make evidence syntheses more streamlined, less duplicative, and more importantly, they may be more useful for timely policy and clinical decision-making; however, that will only be the case if they are rigorously reported and conducted.

We look forward to others’ ideas and proposals for the advancement of methods for evidence syntheses. For now, we encourage dissemination and uptake of the currently accepted best tools and practices for their development and evaluation; at the same time, we stress that uptake of appraisal tools, checklists, and software programs cannot substitute for proper education in the methodology of evidence syntheses and meta-analysis. Authors, peer reviewers, and editors must strive to make accurate and reliable contributions to the present evidence knowledge base; online alerts, upcoming technology, and accessible education may make this more feasible than ever before. Our intention is to improve the trustworthiness of evidence syntheses across disciplines, topics, and types of evidence syntheses. All of us must continue to study, teach, and act cooperatively for that to happen.

## Acknowledgments

Michelle Oakman Hayes for her assistance with the graphics, Mike Clarke for his willingness to answer our seemingly arbitrary questions, and Bernard Dan for his encouragement of this project.

## Funding

JPAI has been supported by an unrestricted gift from Sue and Bob O’Donnell to Stanford University.

## Author contributions

All authors contributed to the development of the ideas, writing, and the final review of the submitted manuscript.

## Supplementary Material

SUPPLEMENTARY MATERIAL
