# Supplementary material for: Guidance to best tools and practices for systematic reviews
Source: JBI Evid Synth. 2023 Jun 7;21(9):1699–731. doi: 10.11124/JBIES-23-00139 (PMC10464882; doi:10.11124/JBIES-23-00139)
Supplement: SUPPLEMENTARY MATERIAL [file srx-21-1699-s004.pdf]

Supplemental File 5A: Illustrations of the GRADE approach

Table SF5A-1: The outcome-centric approach in GRADE

Source	PICO question	Outcomes evaluated[a]	Outcome importance[b]	Systematic review	Recommendation formulation
				Certainty of a body of evidence[c]	Overall certainty of evidence[d]
Hanson 2019[1] and Crocket 2012[2]	Should lubiprostone be used in the management of opioid-induced constipation in patients with non-cancer pain?	Spontaneous bowel movement response	Critical	Low ⊕⊕◯◯	Low
		Reduction in severity of straining	Important	Moderate ⊕⊕⊕◯	
		Adverse effects leading to discontinuation of treatment	Important	Moderate ⊕⊕⊕◯	
Lantos 2021[3]	In patients following a high-risk tick bite, should prophylactic antibiotic therapy be used versus observation?	Clinical evidence of Lyme disease after treatment	Critical	Moderate ⊕⊕⊕◯	Moderate
		Seroconversion	Important	Low ⊕⊕◯◯	
		Serious adverse events	Important	Moderate ⊕⊕⊕◯	

[a] See original citations for a complete list of outcomes that are reported in the GRADE evidence profiles.
[b] Multiple stakeholders participate in defining which outcomes are critical and important. This is an early step in the process for developing clinical recommendations.
[c] Determined by consideration of factors that affect confidence in an estimate of effect.[4] See Table 5.1 in main text for the specific reasons for upgrading and downgrading the certainty of evidence.
[d] For recommendations, overall certainty across outcomes is determined by the lowest certainty of evidence for any outcome rated as critical.[5]

Table SF5A-2: Statement of conclusions on evidence certainty reached with and without application of GRADE[a]

Topic of interest: Should treatment (X) be used to treat (condition) in (population)?	
Critical outcomes: Quality of Life (QoL), significant adverse events (AEs)	
Systematic review research question: Is treatment X more effective than usual care for improving QoL in (condition)?	
**Without application of GRADE**	**With application of GRADE**
Treatment X compared to usual care leads to statistically significant improvements in QoL.	There is (*high, moderate, low, very low*) certainty evidence that Treatment X compared to usual care improves QoL in (population) with (condition).
Treatment X was not associated with significant AEs compared to usual care.	There is (*high, moderate, low, very low*) certainty evidence that Treatment X does not cause more frequent AEs compared to usual care in (population) with (condition).
There is sufficient evidence to suggest Treatment X over usual care for improving QoL in (condition).	There is (*high, moderate, low, very low*) certainty evidence that Treatment X is more effective for improving QoL compared to usual care in (population) with (condition).

[a] The example is a hypothetical systematic review. Adapted from Samuniak and colleagues.[6]

References

1. Hanson B, Siddique SM, Scarlett Y, Sultan S. American Gastroenterological Association Institute technical review on the medical management of opioid-induced constipation. Gastroenterology. 2019;156(1):229-253.e5.
2. Crockett SD, Greer KB, Heidelbaugh JJ, Falck-Ytter Y, Hanson BJ, Sultan S. American Gastroenterological Association Institute guideline on the medical management of opioid-induced constipation. Gastroenterology. 2019;156(1):218–26.
3. Lantos PM, Rumbaugh J, Bockenstedt LK, Falck-Ytter YT, Aguero-Rosenfeld ME, Auwaerter PG, et al. Clinical practice guidelines by the Infectious Diseases Society of America (IDSA), American Academy of Neurology (AAN), and American College of Rheumatology (ACR): 2020 guidelines for the prevention, diagnosis and treatment of lyme disease. Clin Infect Dis. 2021;72(1):e1–48.
4. Guyatt G, Oxman AD, Sultan S, Brozek J, Glasziou P, Alonso-Coello P, et al. GRADE guidelines: 11. Making an overall rating of confidence in effect estimates for a single outcome and for all outcomes. J Clin Epidemiol. 2013;66(2):151–7.
5. Schünemann H, Brozek J, Guyatt G, Andrew Oxman. Section 5.4 Overall quality of evidence. Handbook for grading the quality of evidence and the strength of recommendations using the GRADE approach. GRADE; 2013 [cited 2022 Mar 25]. Available from: https://gdt.gradepro.org/app/handbook/handbook.html#h.lr8e9vq954a.
6. Samuniak D, Watts C, Cumpston M, Lasserson T, Livingstone N, Opiyo N. Common errors: a resource for Cochrane Editors. Cochrane; 2016 [cited 2022 Mar 5]. Available from: https://training.cochrane.org/common-errors.
